# Phase 1b/2a, 6‐month, randomized, double‐blind, placebo‐controlled study of the safety/tolerability and preliminary evidence of efficacy of a tau‐targeted therapeutic in patients with corticobasal syndrome

**DOI:** 10.1002/alz70861_108915

**Published:** 2025-12-23

**Authors:** Lawren VandeVrede, Rebecca Snell, Hilary W. Heuer, Courtney Lane‐Donovan, Peter A. Ljubenkov, Bruce L. Miller, Julio C. Rojas, Adam L. Boxer

**Affiliations:** ^1^ Memory and Aging Center, University of California San Francisco, San Francisco, CA USA; ^2^ Memory and Aging Center, Weill Institute for Neurosciences, University of California, San Francisco, San Francisco, CA USA; ^3^ UCSF, San Francisco, CA USA; ^4^ Memory and Aging Center, UCSF Weill Institute for Neurosciences, University of California, San Francisco, San Francisco, CA USA; ^5^ Weill Institute for Neurosciences and Memory and Aging Center, Department of Neurology, University of California, San Francisco, CA USA; ^6^ Global Brain Health Institute (GBHI), University of California San Francisco (UCSF); & Trinity College Dublin, San Francisco, CA USA; ^7^ University of California, San Francisco, San Francisco, CA USA; ^8^ Memory and Aging Center, Department of Neurology, Weill Institute for Neurosciences, University of California, San Francisco, San Francisco, CA USA

## Abstract

**Background:**

Corticobasal syndrome (CBS) is a devastating and untreatable neurodegenerative disease that interferes with cognition, movement, and behavior, and only a handful of clinical trials have been conducted in this population.^1^ CBS is predominantly caused by tau, but two different types of tau can accumulate: 4R‐tauopathy (CBS‐4RT) or the tau in Alzheimer’s disease (CBS‐AD).^2^ Recently, we demonstrated that a tau blood test (*p* ‐tau217) can distinguish between CBS‐AD and CBS‐4RT,^3^ important because tauopathies may respond differently to therapeutics.^4^ This precision diagnosis permits diagnostic refinement of CBS in clinical trials.

**Methods:**

The primary objective is the safety/tolerability of 6 months of a tau‐directed therapeutic. The secondary objective is to evaluate target engagement by measuring tau in CSF and plasma. The exploratory objective is evaluation of clinical efficacy using clinical, fluid, and imaging endpoints. A total of N=24 participants with a clinical diagnosis of CBS will be stratified with plasma *p* ‐tau217 (goal n=12 per arm) and randomized 2:1 to a tau‐directed therapeutics. The study drug will be administered for 6 months, and safety/tolerability, target engagement, and clinical efficacy will be tested in both a primary (CBS‐4RT) and a secondary tauopathy (CBS‐AD). The current study design calls for a single site with an expected launch date in 2025, with an additional 6‐month open‐label extension.

**Results:**

This trial design represents the first “within‐syndrome” basket design in CBS using precision diagnosis to stratify by neuropathological etiology using the plasma *p* ‐tau217 biomarker; the first use of a tau‐directed therapeutic simultaneously in both an AD and non‐AD tauopathy in a single trial; and the first use of several novel fluid biomarkers of tau engagement (e.g. MTBR‐tau275/282).

**Conclusion:**

A stratified “basket trial” design is a viable mechanism to enroll clinical sub‐cohorts of corticobasal syndrome to test tau‐directed therapeutics in this population.

